# Quantitation of Alzheimer's disease biomarkers in plasma using SMC^®^ high sensitivity immunoassays

**DOI:** 10.1002/alz70856_101217

**Published:** 2025-12-24

**Authors:** Joseph Hwang, Raad Gitan, Deborah Droll, Elizabeth Adkisson, Rick Wiese

**Affiliations:** ^1^ MilliporeSigma, St. Louis, MO, USA

## Abstract

**Background:**

Alzheimer's Disease (AD) affect millions worldwide and is becoming more prevalent as the population ages. Current understanding of AD pathology centers around monitoring neurodegenerative biomarkers in blood. In recent years there specifically has been a growing interest in measuring the ratio of Aβ42/ Aβ40 but these low abundant biomarkers require higher sensitivity immunoassays for detection in blood samples.

**Method:**

We developed and verified a novel SMC^®^ Aβ42/Aβ40 high sensitivity immunoassay kit that can accurately quantitate these biomarkers simultaneously in human plasma samples using FemtoQuest™, the next generation SMC^®^ instrument. Here we report the results from testing plasma samples from healthy and AD patients using the new immunoassay and instrument.

**Result:**

First, SMC^®^ Aβ42 and Aβ40 high sensitivity singleplex immunoassays were used to confirm detectable levels of Aβ42 and Aβ40 in plasma. Each singleplex assay identified a clear differentiation between healthy and AD samples with *p*‐value of <0.05. The same set of healthy and AD samples was subsequently tested with the multiplex SMC^®^ Aβ42/Aβ40 high sensitivity immunoassay to simultaneously measure both biomarkers. The Aβ42/Aβ40 ratio resulted in a stronger differentiation of healthy vs AD than individual biomarkers.

**Conclusion:**

This study demonstrates the value of using multiplex high sensitivity technology to evaluate multiple biomarkers in the same sample. The SMC^®^ Aβ42/Aβ40 high sensitivity immunoassay kit serves as a powerful non‐invasive biomarker tool for monitoring the progression of neurodegenerative diseases such as Alzheimer's disease while saving time and sample. Further studies into other combinations of AD biomarkers will determine if their ratios would also serve as valuable measures of AD related neurodegeneration.

